# Correction to: Safety, Tolerability, and Immunogenicity of Revaccination With mRNA-1345 RSV Vaccine Administered 24 Months Following a Primary Dose in Adults ≥60 Years of Age

**DOI:** 10.1093/infdis/jiag422

**Published:** 2026-08-14

This is a correction to: Mihir Desai, Gilberto Jimenez, Priyantha Wijewardane, Lan Lan, Lauren Wilson, Shraddha Mehta, Frances Priddy, Safety, Tolerability, and Immunogenicity of Revaccination With mRNA-1345 RSV Vaccine Administered 24 Months Following a Primary Dose in Adults ≥60 Years of Age, *The Journal of Infectious Diseases*, 2026; jiag350, https://doi.org/10.1093/infdis/jiag350

The following change has been made to the originally published paper.

In section **Participants**, second paragraph, second sentence text is corrected to read: “[…] and 32.2% (321/998) had one or more comorbidities of interest.” instead of: “[…] and 32.3% (321/998) had one or more comorbidities of interest.”

